# Phase-Dependent Deep Brain Stimulation: A Review

**DOI:** 10.3390/brainsci11040414

**Published:** 2021-03-25

**Authors:** Lekshmy Sudha Kumari, Abbas Z. Kouzani

**Affiliations:** School of Engineering, Deakin University, Geelong, VIC 3216, Australia; lsudhakumari@deakin.edu.au

**Keywords:** brain stimulation, phase-specific brain stimulation, neural oscillations

## Abstract

Neural oscillations are repetitive patterns of neural activity in the central nervous systems. Oscillations of the neurons in different frequency bands are evident in electroencephalograms and local field potential measurements. These oscillations are understood to be one of the key mechanisms for carrying out normal functioning of the brain. Abnormality in any of these frequency bands of oscillations can lead to impairments in different cognitive and memory functions leading to different pathological conditions of the nervous system. However, the exact role of these neural oscillations in establishing various brain functions is still under investigation. Closed loop deep brain stimulation paradigms with neural oscillations as biomarkers could be used as a mechanism to understand the function of these oscillations. For making use of the neural oscillations as biomarkers to manipulate the frequency band of the oscillation, phase of the oscillation, and stimulation signal are of importance. This paper reviews recent trends in deep brain stimulation systems and their non-invasive counterparts, in the use of phase specific stimulation to manipulate individual neural oscillations. In particular, the paper reviews the methods adopted in different brain stimulation systems and devices for stimulating at a definite phase to further optimize closed loop brain stimulation strategies.

## 1. Introduction

Brain stimulation paradigms including electrical deep brain stimulation (DBS), optogenetics brain stimulation (OBS), transcranial electrical stimulation (tES), and transcranial magnetic stimulation (TMS) and repetitive transcranial magnetic stimulation (rTMS) are being increasingly employed as a therapeutic and diagnostic tools for neurological disease conditions such as movement disorders and psychiatric illnesses [1,2,3,4,5]. DBS and OBS are invasive brain stimulation paradigms, where the stimulation electrode is implanted into the brain, whereas tES and TMS are non-invasive brain stimulation (NIBS) techniques in which the brain is stimulated without implanting any devices (e.g., electrode) inside the body [6,7]. Recently, non-invasive techniques such as rTMS are being increasingly used in the treatment of major depressive disorder (MDD) [8], and as a positive development, high efficacy is being also reported in the treatment of depression, pain, and stroke [9]. NIBS is also being studied as a promising treatment methodology for addictions and substance-use disorders (SUDS) [10,11,12]. On the other hand, the invasive technique of DBS is the commonly used in the treatment of common neurological diseases like Parkinson’s disease (PD), epilepsy, tremor, etc. [13,14].

Over the recent years, there have been many advancements in invasive brain stimulation paradigms with size-reduced, tether-less and low power devices. Most of the existing brain stimulation devices in clinical therapeutical use are ‘open-loop’, where the stimulation settings are fixed and subsequent adjustments to the device settings are done manually during hospital visits [15]. However, this open-loop approach limits the efficacy of brain stimulation modalities [16]. One of the main characteristics of neurological disorders is that the associated symptoms change in time and are often progressive [17]. Due to these fluctuating symptoms, often the efficacy of the system reduces with time [18]. Adding to this, some of the other limitations of the stimulation include introducing disabling side-effects, not significantly affecting some of the disease symptoms, manual programming upon frequent symptom assessment, etc. [19]. All these limitations could be attributed to the inherent-property of fixed stimulation settings and stimulation regardless of the state of the individual in open-loop stimulation. Hence an automated approach is desirable. This can be achieved in a closed-loop approach in which the stimulation parameters can be adjusted in real-time depending on a feedback signal from the subject, thus making it possible for changing the stimulation based on the fluctuating symptoms and further paving the way for patient-tailored treatment for neurological disorders. A closed loop stimulation is realized by sensing an individual’s brain signals and using it as the feedback signal to the stimulation circuit. Finally, this feedback signal could help in accurately adjusting the stimulation parameters for better control of disease symptom with lesser side effects [20]. Promisingly, various studies on PD with closed loop DBS have presented good results. For example, Arlotti et al. [21] assessed closed-loop DBS of subjects with PD, reporting the stimulation as a safe and effective treatment practice for PD. In another work, Swann et al., [22] demonstrated the efficacy and feasibility of closed loop DBS with fully implanted devices in PD patients. Similar studies demonstrate the high efficacy of closed-loop DBS [23,24,25]. Recently, the United States Food and Drug Administration has approved Medtronic Percept, the first commercially available DBS system that can record brain activity while simultaneously stimulating, to treat PD symptoms [26].

For the development of closed-loop or adaptive brain stimulation strategies, the feedback signal is generated from the subject by reading different biomarkers [27] that reflect the disease state of the subject. Establishing accurate biomarkers for disease states is crucial for increasing the efficacy of the closed loop brain stimulation systems. Biomarkers in the existing brain stimulation devices and systems are of two types: electrophysiological and neurochemical. While neurochemical biomarkers indicate the state of neurotransmitters [28], electrophysiological biomarkers (e.g., action potential (AP) as well as local field potential (LFP)) provide electrical activity of the brain. The action potential or high frequency neural spike is the fundamental method of communication between neurons, and hence is considered an important signal for understanding the underlying neurological conditions. Action potential is measured invasively using microelectrodes followed by high pass filtering of the signal [29], whereas, local field potential reflects the combined electrical activity of a group of adjacent neurons and is measured from the invasively recorded extracellular electrophysiological activity by low pass filtering around 200 Hz [30]. Analyzing LFP is like interpreting rhythmic brain action from electroencephalography (EEG) recordings. Moreover, as the electric field generated by the nerve cells are subject to an exponential decay with distance, producing a detectable signal that needs a smaller number of nerve cells to be simultaneously active in LFP than in EEG which is recorded non-invasively [31].

The repetitive patterns in the neural activity are observed in LFP as oscillations. These neural oscillations or rhythms are produced by multiple neurons communicating with each other and for allowing synchronized action during normal brain operation. Power or amplitude of these neural oscillations are related to different cognitive functions [32], and alterations in these oscillations is linked to the neural underpinnings of different neurological diseases. In order to investigate these neural oscillations and deepen our understanding of various neurological conditions, it is desirable to have an efficient closed loop brain stimulation system capable of measuring and decoding the amplitude of a particular band of oscillation and control the stimulation parameters based on these oscillation properties. This will very well serve the therapeutical purpose of brain stimulation. Additionally, by utilizing stimulation techniques like optogenetics [33], where precise control of neural circuits using a specific wavelength of light, closed loop brain stimulation could even be used for investigating the causative mechanism of neurological disorders. For example, Piantadosi et al. [34] employed closed loop optogenetics brain stimulation to understand the role of cortico–stratial–thalamo–cortical (CSTC) neural circuits and the obsessive–compulsive disorder (OCD) in animals. This will serve both therapeutical and diagnostic purpose of the brain stimulation. However, a major challenge in implementing such a design is delivering the neural stimulation to alter the power or amplitude of one particular band of oscillation in the neural signal. This is where the concept called phase-specific brain stimulation becomes the key.

Generally, in an ON-OFF feedback control strategy, stimulation pulses are delivered when the amplitude/power of the oscillation under consideration deviates from a certain threshold value. Dual-threshold strategies can also be found in literature where the stimulation voltage is either increased or decreased based on the upper and lower range of oscillatory band power [24]. In either case, only the amplitude/power of the oscillation is considered for delivering the stimulation pulses. On the contrary, in phase-specific brain stimulation, the stimulation signal is applied considering both the threshold as well as the instantaneous phase of the neural oscillation under observation. By taking both the amplitude and phase of the LFP signals into consideration, a better method for manipulating specific frequency bands in the neural oscillations could be achieved.

In the following sections, neural oscillations, the importance of neural oscillations as biomarkers for neurological diseases, and significance of phase selective close-loop brain stimulation strategies are discussed. Some of the current research work involving the strategies being used by studies for implementing stimulation in correct phase are explained. These works support the expectation that phase-specific closed loop brain stimulation will demonstrate higher efficacy in modulating neural pathways.

## 2. Neural Oscillations as Biomarkers

Neural oscillations are the repetitive electrical activity generated spontaneously or in response to stimuli by neurons [35]. Oscillatory activity of the neural assemblies can be categorized as delta (0.5–3.5 Hz), theta (3.5–7 Hz), alpha (8–13 Hz), beta (18–25 Hz), gamma (30–100 Hz), and high-frequency oscillations, HFO (100–200 Hz). There is extensive evidence to suggest that these neural oscillations and the synchronization between these neural oscillations in various cortical regions help in establishing different cognitive phenomenon and memory functions. In addition to their role in normal brain functioning, studies have suggested the alterations in alpha, beta, gamma, delta, and theta frequency band activities may be associated with different neuropsychiatric disorders. These neural oscillations and their synchrony help in establishing different cognitive phenomenon and memory functions [36,37]. Studies have suggested the modulations in alpha, beta, gamma, delta, and theta frequency bands in pathological brains [38,39,40]. Features of the neural oscillations like location, amplitude, frequency, and phase are significant [41] and determine its effect on the neural pathway. There exist research work suggesting changes in oscillatory dynamics in common conditions such as major depressive disorders (MDD), PD, AD, epilepsy, Schizophrenia (SZ), and so on. Additionally, it is important to note that each function in the brain is the result of combined actions of multiple oscillations [42], which makes finding the apt biomarkers for these brain conditions based on changes in neural oscillations a very complex problem to decode. In this context, a closed loop brain stimulation system based on oscillation-related biomarkers provides a good opportunity to come up with understanding of the function of the neural oscillations, and better elucidate these cognitive disorders, their progression, and effects of medicines on the neural disorders.

Considering one of the most common neurodegenerative motor impairment diseases, PD, the role of modulations in beta band oscillations in basal ganglia- cortical circuits has been widely studied [43,44,45,46,47]. These studies suggest a direct correlation of reduction in beta band power to bradykinesia, the motor impairment in PD. A general agreement in all these studies is the fact that a high beta band power may contribute to motor impairment in PD, and thus a reduction in beta band power could lead to clinical improvement in PD [15,46]. Along with beta band oscillations, there are findings that suggest a role for broad band gamma oscillations in the range of 50 to 200 Hz in motor dysfunctions in parkinsonian state. Studies have reported the increased activity in motor cortex resting broad band gamma [48] along with exaggerated cross-frequency coupling of broadband gamma to the phase of the beta rhythm [49,50,51]. Özkurt et al. [52] reported the interaction between high frequency oscillations bands around 250 and 350 Hz in the subthalamic nucleus (STN) in pathophysiology of PD. A positive correlation of theta activity in STN and negative correlation with STN beta activity with rest tremor in PD is reported in [53]. Other than the movement disorder, there are several non-motor symptoms like cognitive impairments and neuropsychiatric symptoms, and sleep disorders, among others associated with PD [54,55]. Attention difficulties are characteristics of cognitive impairment in PD. Bin Yoo et al. [56] discussed enhanced activity in bilateral gamma in a bottom-up attention stream and increased left alpha2 (10–12 Hz) connectivity in the top-down attention stream. The study also reported a higher alpha2-gamma coupling in the right posterior parietal cortex in PD patients than in the healthy subjects under study.

Dystonia [57,58] is yet another movement disorder caused by dysfunction of brain regions and the communication between neurons and involves involuntary muscle contractions. Pallidal DBS is being used for the treatment of dystonia [59]. However, therapeutic mechanism of DBS, and a proper biomarker for dystonia are still under study. Some studies, e.g., [60,61,62], have suggested enhanced internal pallidum theta band activity having robust association with symptoms in cervical dystonia (CD) [63], and in [61] theta band oscillation is suggested as a possible biomarker for closed-loop brain stimulation in CD.

Talking about the debilitating neuropsychiatric disease, SZ, there is still more to be understood about the origin and progression of the disease and hence a definite biomarker for early detection and diagnosis of the disease is still under study [64,65]. Along with other hypothesis put forward by different researchers, the role of abnormalities in neural oscillations are also highlighted as the underlying mechanism for symptoms associated with SZ. Synchronous gamma band activity is observed to be the mechanism for facilitation of sensory and cognitive processes in healthy humans [66], and hence gamma oscillation properties could function as a biomarker for the diagnosis of SZ as the defining symptoms of SZ include cognitive and perceptual abnormalities. This is supported by the fact that there is mounting evidence suggesting the neural oscillations in the gamma frequency range are modified in SZ [67].

Considering the literature on the common psychiatric illness, major depressive disorder (MDD or clinical depression), abnormal oscillatory patterns in different frequency bands have been widely reported. Among these, the role of alpha band oscillations in MDD can be seen as a consistent finding [68,69,70]. The findings from these studies include an elevated absolute or relative alpha power at parietal, frontal, or occipital sites. Researchers have also reported the role of theta band rhythms in connection with MDD [71]. There is an increasing body of evidence indicating that the lower gamma band oscillatory power can be a prospective biomarker for MDD [72]. In one of the other major neuronal disorders, AD, research points at a decrease in alpha, beta, and gamma oscillatory power over posterior regions and enhancements in resting state delta and theta power [73]. Gamma oscillations are suggested to contribute to memory encoding as well as retrieval, and hence it is not surprising to find alterations in the gamma band oscillation in patients with AD and rodent models of AD [74,75].

With the modulation in neural oscillation being an emerging biomarker for different neurological diseases, a closed loop brain stimulation system with adaptive stimulation according to the online reading of the biomarker is a very good tool to establish the relation between these neural oscillations and different neurological conditions. This will further enable science to decode these disease features for developing medicines and other treatment methodologies. The concept is to read the biomarker continuously and change the stimulation accordingly. By applying the stimulation, the aim is to target the particular neural oscillation frequencies, and this is where phase specific stimulation comes into picture.

## 3. Closed Loop Brain Stimulation with Neural Oscillations as Biomarkers

On the device engineering side, the essential difference between a closed and an open loop control system is in the feedback mechanism. In a closed loop control system, a feedback control is present, which can efficiently change the stimulation parameters depending on the actual and desired output. Using extra circuitry, the biomarkers are quantitatively analyzed to provide precise stimulation at accurate times. This helps address one of the major issues associated with neural disorders, i.e., that the symptoms do no stay constant, and the condition of the patient could vary throughout a period. With the help of an effective closed loop control in place, it can be ensured that the person is given optimal stimulation by continuously monitoring the biomarkers and modulating the stimulation parameters accordingly. Moreover, closed loop brain stimulation, as mentioned before, helps in the diagnosis as well as study of neural disorders. This is important as the scientific community is still investigating the cause of origin and progression of multiple commonly occurring neurological diseases. In addition to this, for untethered devices, extra stimulation uses up battery life unnecessarily.

With neural oscillations as biomarkers, closed loop brain stimulation would be benefitted from phase specificity.

## 4. Phase Specific Stimulation

As pointed out in the previous sections, manipulating the neural oscillations in LFP could prove to be a useful treatment approach for different neural conditions. However, controlling the power of an individual frequency band of oscillations in the LFP is not an easily achievable task [76]. Another important factor to consider while using neural oscillations as biomarkers and high frequency stimulation is the possible side effects. These side effects are mainly caused by the non-specificity in the high frequency stimulation. Stimulation induced side effects are due to the stimulation being not directed specifically to the neurological signals driving the disease symptoms [77]. Hence it is important for the brain stimulation to be more specific to the pathological neural activity.

**Figure 1:**
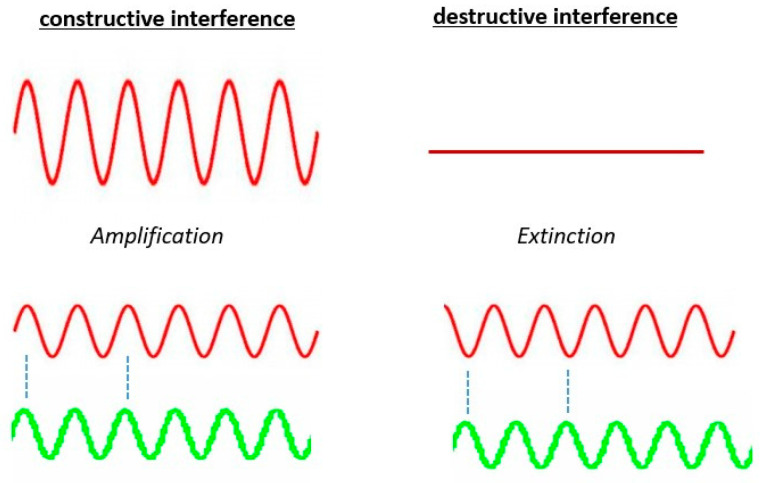
Constructive and destructive interference—concept and illustration.

An approach to enhance the specificity of brain stimulation and modulate the oscillatory power in specific bands of neural oscillations is to time-lock the stimulation to the phase of the present oscillation. Adopting the concept of constructive and destructive interference (Figure 1), stimulating at oscillatory peaks of the desired neural oscillation improves the present oscillation, while stimulating at oscillatory troughs suppresses an ongoing oscillation [78,79]. This can be termed as phase-specific brain stimulation. As this stimulation strategy is specific to a specific neural oscillation, it may cause fewer side-effects as other rhythmic oscillations which are not phase-locked to the stimulation would not be affected. As simple as it may sound, the challenge of realizing such a system lies in accurately predicting the oscillatory phase rapidly and delivering the apt stimulation pulses in real-time in a feedback loop.

Reviewing the literature on brain stimulation systems employing phase-specific stimulation, we came across very few developed systems, including both invasive and non-invasive systems. These systems are further described in the following.

Mansouri et al. [80] discussed a bench-top closed loop transcranial electromagnetic brain stimulation platform that can interpret EEG signals in real time, predict the phase of the underlying brain oscillations, and deliver controlled pulsed transcranial electromagnetic stimulation output at a precise phase of the target neural oscillation. Alpha and theta band of oscillation were the target bands in this work. Phase specificity was introduced in the system and was implemented using an EEG system employing an Arduino development board, and Matlab software. The authors calculated the phase delay introduced by the signal processing to be 3.8 degrees for theta band and 57 degrees for alpha band stimulation. The phase of the incoming EEG signal was determined by considering a portion of the signal followed by the use of a 10th order elliptical filter to filter out the undesired frequencies. After getting the frequency band of interest, the fast Fourier transform (FFT) of the filtered signal was calculated. The FFT bin with the highest power in the desired frequency band was considered to select the dominant frequency. Further, the timing of the next stimulation pulse is computed from the phase and frequency values of the calculated dominant frequency. The authors also considered the minute delays in the system or small phase shifts that could have been introduced in the system by the filter or other signal processing components or hardware delays. These delays in the system was accounted for in the stimulation time by presenting an empirically calculated correction time in phase. For example, the execution time of the Matlab code was approximately calculated to be 1 ms and was added to the pulse time to account for the delay. Stimulation is applied using the transcranial electrical stimulator from Neuroconn. Timing of the stimulation is communicated to the microcontroller in the Arduino board using serial USB communication. Microcontroller generated voltage waveform depending on the timing and is used to control the stimulator which delivers a constant current output relative to the voltage.

Siegle et al. [78] used a phase-locking approach with closed loop optogenetic brain stimulation in freely behaving mice to cause inhibition of dorsal hippocampal CA1 at specific phases of theta band oscillations. The study reported an improved performance when the stimulation introduced in the encoding segment was triggered by the maxima of theta rhythm. The LFP signal was sensed using electrodes and digitally filtered between 4 and 12 Hz to record the theta band oscillations. Optogenetic stimulation was activated once the theta power passed a threshold. Once the sensed signal was at a local minimum or maximum, the control algorithm directed the optogenetic stimulator to deliver a 10 ms light pulse to the invasively implanted fiber optic electrode. The study reported an average delay between trigger and the beginning of the light pulse of 21.7 ± 7.2 ms for peak-activated stimulation and 21.3 ± 7.4 ms for trough-activated stimulation. Accordingly, the reported mean phase of stimulation was 96 ± 54° for peak-activated stimulation and −131 ± 63° for trough-activated stimulation. The phase-based trough-activated stimulation demonstrated lower precision. Another point to note is that the stimulation was 90–180 degrees away from the target phase.

In another related work on phase-specific deep brain stimulation for movement disorder, Cagnan et al. [81], considered the dominant phase of tremor in subjects with essential tremor. The signals from an accelerometer were analyzed using Matlab software. Firstly, the signals were band-pass filtered at ± 2 Hz around the tremor frequency using 4th order Butterworth filter, to get the tremor signals. Amplitude and instantaneous phase of the tremor frequencies was calculated using Hilbert transform. Low frequency phase locked stimulation was given at random phase with respect to the tremor phase, and the best phase-offset was determined by trial and error. Phase-locked DBS was then introduced during tremor episode. A symptom suppression of up to 87% was reported. Along with increase in efficacy, by using low-frequency phase-locked stimulations, the authors were able to power savings in the system.

Zarubin et al. [82] used a closed-loop transcranial alternating current stimulation system for understanding the effects of repetitive short-time visual cortex stimulation on the amplitude of visual alpha band oscillation when the stimulation was modified based on the frequency and phase of those alpha band oscillations. EEG signals were used for the experiment. For the phase prediction Hilbert transform based approach was used. In order to reduce the complexity, quasi-stationary nature of phase dynamics for the short time interval was assumed. For predicting the phase-dependent stimulation, the phase information of the last 250 ms of the ongoing interval is used considering the quasi-stationary nature. Initially, FIR filtering is employed to get the alpha band oscillations. This was followed by Hilbert transformation to obtain instantaneous phase values. Phase prediction was optimized by repeated search over sine waves of diverse phases. For this, Euclidean difference (L2 norm) for vectors of immediate phase between extraction interval and several produced sine waves was done. Further, delay due to this optimization was calculated and compensated.

In another important work in this area, an analog feedback circuit is used to give phase-specific stimulation. In reference [76], a closed loop transcranial electrical stimulation is explained, where phase locked stimulation was used to enhance only the alpha oscillations (8–15 Hz) of the subject. The closed loop circuit was realized in analog feedback circuit instead of the usually used digital circuits. The front-end circuit, which acquires the neural signals from the subject, consisted of a multiple feedback filter realized using the Linear Technology LT1012 operational amplifier IC and related passive components. The front end efficiently receives the signal and filter out the alpha band frequencies form the signal. A pre-amplifier stage is realized using a Cereplex digitizing amplifying head stage to give the required amplification to the filtered out alpha band signals. As the following stage is an analog feedback circuit, the digital output from Cereplex is converted to analog using a signal processor unit by Cerebus before feeding to the analog circuit. The analog feedback circuit is followed by a transcranial electrical stimulator by Neuroconn GmbH. The feedback circuit’s output controls the stimulator output current with a rate of 2 mA per applied voltage. The calculated system delay contributed by the analog amplification circuit and the digital components is 372° at 12 Hz and 360° at 11.6 Hz. Thus, the delay creates a positive feedback loop for alpha band frequency amplification in the neural signal by providing closely in-phase electrical stimulation using the Neuroconn stimulator. The advantage of the design is that it can be easily modified for other frequency band of neural oscillation by changing the filter design for a different passband and the total loop delay. However, on the hardware side, for the implementation of the analog feedback circuit, the digitized signal is converted back to analog, which requires extra circuitry.

While reviewing these systems, it is clear that these systems lack a dedicated circuit for the phase detection and phase specific stimulation. Most of the reviewed systems work in a bench-top setting and the software for phase detection is implemented on a separate PC. This could be a hindrance to the miniaturization and further tether-less operation of brain stimulation systems and there is scope of improvement which could be the development of application specific integrated circuits specifically for the phase detection and precise phase-specific stimulation.

## 5. Outlook: Closed Loop Brain Stimulation with Phase Specific Stimulation

**Figure 2:**
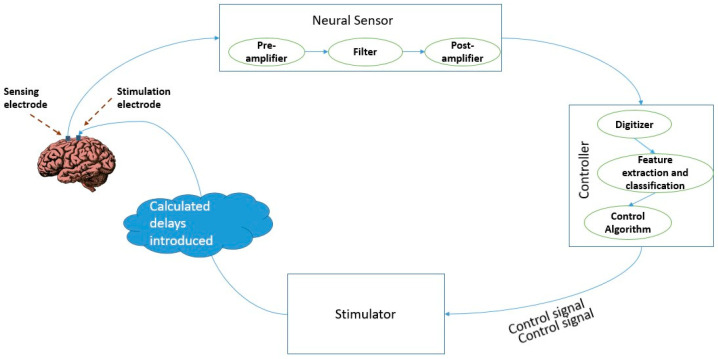
Block diagram of phase specific deep brain stimulation.

The essential components of a closed loop brain stimulation system with phase specific stimulation for LFP-based biomarkers are the following: a neural sensor to sense the LFP signals from the subject, a neural stimulator for producing the stimulation patterns in the required specifications at the right time, and a software unit to process the signals received by the sensor and generate a control signal for the stimulator. A conceptual block diagram of such a device is presented in Figure 2. The essential stages can be listed as: neural sensing, feature extraction, classification, control, and stimulation.

Electrophysiological signals in the brain are sensed using electrodes invasively implanted into the patient’s brain. A sensor circuit, comprising amplifiers and filters, conditions the sensed signal for further analysis. Initially, the micro amplitude range signals from the electrode are amplified. This is followed by a band pass filter to filter out unwanted frequency signals. The cut off frequency of the band pass filter is adjusted to meet the frequency range of the neural oscillation bands—delta (0.5–3.5 Hz), theta (3.5–7 Hz), alpha (8–13 Hz), beta (18–25 Hz), gamma (30–100 Hz), and high frequency oscillations, HFO (100–200 Hz). This is followed by a controller which extracts the features of the conditioned signals after digitizing them. Features such as amplitude, phase, and power of the received signals are extracted and based on the extracted features, signals are classified into classes.

Following this, a control signal is generated for closing the feedback loop. A control algorithm identifies deviations, if any, of the actual stimulation output from the required level of stimulation. Accordingly, the algorithm adjusts the stimulation parameters to minimize the difference in actual and desired outputs with the help of the stimulation control circuit. Thus, the algorithms and software module for real time classification of the LFP signals into pathological and non-pathological also have a very important role to play in the realization of an efficient closed-loop brain stimulation system. The actual clinical implementation of closed-loop DBS for rapid behavioral changes relies on accurate and quick detection of these disease states using efficient algorithms. Algorithms ranging from very simple classification algorithms to sophisticated machine learning and artificial intelligence-based ones can be found in literature.

While adding the attribute of phase specificity in the stimulation to the device, the controller needs to have the extra feature of calculating the phase of the ongoing signal oscillations, and the delays in the network to produce the control signal for the stimulator to send the correct amplitude pulse at the right time and phase to the stimulation electrode placed on the brain surface. Thus, the most significant requirement in the realization of such a phase-specific deep brain stimulation is the implementation of an algorithm for real time determination of the phase of the uninterruptedly recorded local field potential information. A key difficulty in such a system is accurately estimating the oscillatory phase in real time. An efficient algorithm to control the stimulation pulse and trigger the stimulation at the right time considering the phase of the ongoing stimulation as well as the delays along the network needs to be realized. In the existing digital signal processing systems, by the time a signal is digitized and its phase is computed through a Fourier transform based algorithm, the target phase has long gone. A quicker but complex method involves outputting phase-locked stimulations using analog-circuitry which is still in the early stages of development. Additionally, such an analog circuit can be implemented into an application specific integrated circuit chip.

Another important factor to consider for potentially optimizing brain stimulation is the functional connectivity of the brain. It is a well-understood fact that brain regions don’t operate in isolation. Our brain is a network, which consists of spatially distributed, but connected sections that work together in realizing different function [83,84]. Hence the connectivity between the point of stimulation and other sections of the brain could greatly affect the efficiency of DBS treatment for neurological conditions [85]. Horn et al. [86] developed a structural and functional connectivity profile for effective STN DBS for PD to predict the outcome efficacy of DBS. On a similar note, Fox et al. [87] predicted the efficacy of TMS in depression by considering the functional connectivity. Hence, it can be concluded that electrode placement is highly important in the clinical success of brain stimulation methodologies, among others.

## 6. Conclusions

Phase selective stimulation is an added attribute to the deep brain stimulation paradigm for increasing its efficacy in altering the neural oscillations in specific frequency bands. As more and more studies are being carried out to find the link between the variations in the energy of different frequency bands of neural oscillations and neurological disorders, a hybrid strategy combining closed-loop DBS strategies and phase selective stimulation could be an effective approach. Selective modulation of neural synchrony using phase-locked stimulation offers the potential to enhance the efficacy of therapy and minimize the side effects of the stimulation. While reviewing the literature on this approach, the authors could not find much work done using phase selective stimulation in invasive DBS. This paper reviewed the methodologies adopted in different brain stimulation systems and devices for stimulating brain at a definite phase. There is also an attempt to put forward a conceptual model incorporating the concepts of phase selective stimulation and deep brain stimulation for standalone DBS devices.

## Data Availability

Not applicable.
